# Impact of Natural Compounds on DNA Methylation Levels of the Tumor Suppressor Gene *RASSF1A* in Cancer

**DOI:** 10.3390/ijms18102160

**Published:** 2017-10-17

**Authors:** Reinhard H. Dammann, Antje M. Richter, Adriana P. Jiménez, Michelle Woods, Miriam Küster, Chamindri Witharana

**Affiliations:** 1 Institute for Genetics, Justus-Liebig-University Giessen, D-35392 Giessen, Germany; antje.m.richter@gen.bio.uni-giessen.de (A.M.R.); adriana.jimenez@gen.bio.uni-giessen.de (A.P.J.); michelle.woods@bio.uni-giessen.de (M.W.); miriam.kuester@gen.bio.uni-giessen.de (M.K.); 2 German Center for Lung Research (DZL), Universities of Giessen and Marburg Lung Center, D-35392 Giessen, Germany; 3 Department of Biochemistry and Molecular Biology, Faculty of Medicine, University of Colombo, Colombo 0080, Sri Lanka; chamindri@bmb.cmb.ac.lk

**Keywords:** DNA methylation, tumor suppressor gene, *RASSF1*, demethylation, natural compounds

## Abstract

Epigenetic inactivation of tumor suppressor genes (TSG) is a fundamental event in the pathogenesis of human cancer. This silencing is accomplished by aberrant chromatin modifications including DNA hypermethylation of the gene promoter. One of the most frequently hypermethylated TSG in human cancer is the *Ras Association Domain Family 1A* (*RASSF1A*) gene. Aberrant methylation of *RASSF1A* has been reported in melanoma, sarcoma and carcinoma of different tissues. *RASSF1A* hypermethylation has been correlated with tumor progression and poor prognosis. Reactivation of epigenetically silenced TSG has been suggested as a therapy in cancer treatment. In particular, natural compounds isolated from herbal extracts have been tested for their capacity to induce *RASSF1A* in cancer cells, through demethylation. Here, we review the treatment of cancer cells with natural supplements (e.g., methyl donors, vitamins and polyphenols) that have been utilized to revert or prevent the epigenetic silencing of *RASSF1A*. Moreover, we specify pathways that were involved in *RASSF1A* reactivation. Several of these compounds (e.g., reseveratol and curcumin) act by inhibiting the activity or expression of DNA methyltransferases and reactive *RASSF1A* in cancer. Thus natural compounds could serve as important agents in tumor prevention or cancer therapy. However, the exact epigenetic reactivation mechanism is still under investigation.

## 1. Introduction

DNA methylation is an important epigenetic modification in mammalians and occurs predominately at CpG dinucleotides in the genome. At CpG sites, cytosine is modified by an enzyme called DNA methyltransferase (DNMT) and a methyl group is added at the 5-position. De novo methylation is catalyzed by DNMT3A and DNMT3B. In double-stranded DNA, methylated CpGs are short palindromic sequences, and methyl groups which are located in the large grove of the double helix are recognized by methyl-CpG binding domain-containing proteins (e.g., MBD2). These proteins recruit chromatin silencing complexes that result in condensation of the chromosomal region. During semi-conservative DNA replication, unmethylated CpGs on the newly synthesized daughter strand are methylated by DNMT1. This maintenance DNA methyltransferase is recruited to the hemi-methylated CpG sequences with the help of several co-factors including PCNA and UHRF1 [1,2].

In mammalians CpG and GC-rich regions, so-called CpG islands are often localized at regulatory regions like gene promoters and imprinting centers. Unmethylated CpG island promoters are in an open chromatin configuration and are correlated with active gene transcription. However, hypermethylated CpG islands consist of epigenetic inactive chromatin regions. During aging and carcinogenesis, several CpG island promoters become aberrantly methylated. Hypermethylation of tumor suppressor genes (TSG) is a hallmark of their inactivation in the pathogenesis of cancer. One of the most frequently hypermethylated CpG islands of a TSG is located in the promoter region of the *Ras Association Domain Family 1A* (*RASSF1A*) gene [3,4].

## 2. The Ras Association Domain Family 1 (*RASSF1*) Gene

**Figure 1:**
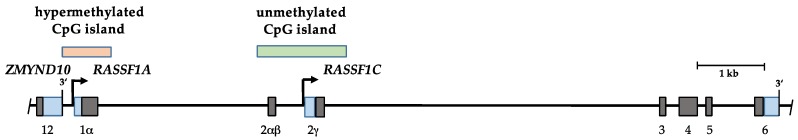
Genomic organization of the *RASSF1* gene. 12 kb of the genomic region of chromosome 3p21.3 (hg19: 50,379,000–50,367,000) is shown. Exons are numbered and depicted as black and blue boxes indicating coding and untranslated regions, respectively. The *RASSF1A* isoform is transcribed from a CpG island containing 84 CpG sites, that is often hypermethylated in cancer. Transcription of *RASSF1C* initiates a downstream located CpG island consisting of 139 CpGs. Transcription start sites are indicated with arrows and transcription end sites are marked with 3′. *ZMYND10* represents the last exon (12) of the *zinc finger MYND-type containing 10* gene, which is located 200 bp upstream of *RASSF1A* promoter.

In lung cancer, deletion of a region in 3p21.3 of chromosome 3 has frequently been reported [5]. Chromosomal deletions of this region have also been found in other cancer entities (in tissues of e.g., breast, head and neck, kidney and gastrointestinal tract) [6]. In the minimal deletion region, we and others have discovered the *Ras Association Domain Family 1* gene (Figure 1) [4,7]. Initially, we termed the gene *Ras effector homologue* 3p21, since its protein sequences exhibited homologies to the murine *novel Ras effector 1* (*Nore1*) [8]. However, the HUGO gene nomenclature committee renamed it to *Ras Association Domain Family 1* (*RASSF1*) gene and human *NORE1* was later renamed *RASSF5*.

**Figure 2:**
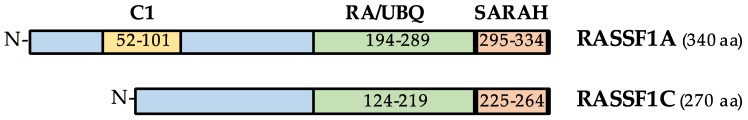
Protein domains of RASSF1A and RASSF1C. RASSF1A is a 340 amino acid (aa) long protein with a protein kinase C conserved region (C1), Ras association/ubiquitin-like (RA/UBQ) domain and Sav/Rassf/Hpo (SARAH) domain. RASSF1C is a 270 aa long protein and encodes a RA/UBQ and a SARAH domain.

Subsequently, we identified two main isoforms of *RASSF1*, that were transcribed by two distinct CpG island promoters: *RASSF1A* and *RASSF1C* (Figure 1). A third variant initially named *RASSF1B* was only expressed at very low levels, and its biological function was not further investigated [4]. The transcript of *RASSF1A* contains six exons (1α, 2αβ, 3, 4, 5 and 6) and is translated to a protein with 340 aa (Figure 2). The *RASSF1C* variant is transcribed from an intragenic CpG island and consists of 5 exons (2γ, 3, 4, 5 and 6). On protein level, *RASSF1A* and *RASSF1C* both encode a C-terminal Ras Association domain (RA) (Figure 2). The rat sarcoma genes (*Ras*) are a family of small GTPases that function as proto-oncogenes by regulating mitogen-induced signaling pathways. In contrast to RASSF5/NORE1, which interacts with several Ras proteins, the direct interaction of RASSF1 with Ras proteins is rather weak [9]. Most likely, the interaction of RASSF1A with Ras is indirect through binding to the endogenous RASSF5 [9]. The RA domain of RASSF1 is also defined as an ubiquitin-like domain (UBQ) [10]. A domain that is only present in the RASSF1A protein is the protein kinase C conserved region 1 (C1) (Figure 2). The C1 domain has been characterized as a binding site for phorbol esters and diacylglycerol, which act as tumor promoters [4].

The *RASSF1* isoforms and *RASSF5* encode a C-terminal SARAH (Sav/Rassf/Hpo) domain that is a characteristic coiled-coil structure (Figure 2). It is a small helical module that is important in signal-transduction networks and serves for protein-protein interactions [11]. This SARAH domain is also found in the regulatory protein WW45 (human homologue of the *Drosophila* protein Salvador/Sav) and the serine/threonine kinase STK3 and STK4 (human homologues of the *Drosophila* kinase Hippo/hpo) [11,12,13]. STK3 and STK4 are also often termed MST1 and MST2 (mammalian STE20-like protein kinase), respectively. Mutation in the *Drosophila Hpo* gene leads to organ overgrowth which was termed the hippopotamus (Hippo) phenotype [14]. In mammals MST1/2 kinases regulate the Hippo pathway through phosphorylation of the large tumor suppressor kinases (LATS) which in turn phosphorylate the transcriptional regulator YAP1 (Yes-associated protein 1) [15]. YAP1 was initially characterized as a protein that interacts with the src-family tyrosine kinase YES1 (homologue of the Yamaguchi sarcoma viral oncogene) and acts as a proto-oncogene [16,17]. Deregulation of the Hippo pathway results in tissue overgrowth and cancer in mammalians. In this signaling network, RASSF1A acts as an upstream activator through its interaction with MST1/2 and WW45 [13,18]. *RASSF1A* is the only tumor suppressor gene that is frequently inactivated in this pathway [19,20,21]. Thus reexpression of RASSF1A induces cell cycle arrest [22,23] and also activates the YAP1 target gene *ANKRD1* [19]. Activation of *ANKRD1* was absent in HeLa cells that harbor an unmethylated *RASSF1A* promoter and express *RASSF1A* [19].

## 3. Hypermethylation of *RASSF1A* in Human Cancers

Initially, we observed that the *RASSF1A* CpG island promoter is frequently hypermethylated in lung cancer [4]. Hypermethylation of *RASSF1A* was revealed not only in non-small cell lung cancer but also in small cell lung cancer [4,24,25,26]. Interestingly, aberrant methylation of the *RASSF1C* CpG island promoter was not observed [4,24]. During the last two decades, it turned out that *RASSF1A* is frequently inactivated in all types of human cancers and promoter methylation of *RASSF1A* has been revealed in cancers from breast [27], liver [28], pancreas [29], prostate [30], renal cell [31,32], brain [33], gastric [34], head and neck [20], pheochromocytoma [35], thyroid [36] and others [31,37,38]. *RASSF1A* hypermethylation is also found in skin cancers including melanoma and Merkel cell carcinoma [39,40,41]. Hypermethylation of *RASSF1A* has been also observed in osteosarcoma and soft tissue sarcomas [42,43]. However, in other cancer entities like cervix carcinoma and colon carcinoma *RASSF1A* hypermethylation is also present but less frequent (<20%) [31,44]. In blood cancer, *RASSF1A* hypermethylation was frequently found in Hodkin’s lymphoma, but was not revealed in chronic myeloid leukemia [45,46]. Interestingly, *RASSF1A* hypermethylation has been correlated with different hallmarks of advanced pathogenesis of cancer, for example with increased progression, advanced staging or metastatic properties [36,47,48]. It has also been reported that *RASSF1A* methylation correlated with a poorer prognosis of cancer patients [42,48]. It has been suggested that *RASSF1A* is one of the most frequently hypermethylated tumor suppressor genes in human cancers and may serve as biomarker for cancer detection [48,49].

Interestingly, we found that normal human mammary epithelial cells, when grown in cell culture for several passages, displayed a senescence-associated hypermethylation of the *RASSF1A* promoter [50]. This indicates that the *RASSF1A* promoter is susceptible to its epigenetic silencing. Recently, we revealed that for tandem-oriented genes, the downstream gene is significantly higher methylated when the transcriptional end site of the upstream gene is in proximity (<1 kb distance) with the transcriptional start site [51]. The *RASSF1A* promoter is located only 200 bp downstream of the last exon of the *ZMYND10* gene (Figure 1). *ZYMND10* encodes a protein containing a MYND-type zinc finger domain, that likely functions in assembly of the dynein motor [52]. In lung cancer, we observed a hypomethylation of the *ZYMND10* promoter and increased expression of its transcript [51]. This data suggest that through its genomic organization *RASSF1A* is prone to epigenetic silencing. Additionally, in cancer we and others observed that aberrant expression of DNMT1, DNMT3A, DNMT3B and histone deacetylases (HDAC) is involved in the inactivation process of *RASSF1A* [51,53,54,55,56].

## 4. Demethylation of *RASSF1A* by Treatment of Cancer Cells with Cytidine Analogues

In contrast to mutated tumor suppressor genes that express aberrant proteins, epigenetically silenced genes are rather infrequently mutated and their reactivation has been postulated as a cure for cancer malignancies [57,58]. Thus, different inhibitors of DNA methylation have been tested, and specifically cytidine analogues are employed in therapy for specific blood cancers [59,60]. In general, these synthetic analogues lead to passive demethylation by inhibiting the methylation of the newly synthetized DNA strand during DNA replication. In contrast to cytidine, which harbors a carbon at position 5 of the base, 5-aza-cytidine and 5-aza-2′-deoxycytidine contain a nitrogen at this position and therefore cannot be methylated by DNMTs. 5-aza-cytidine (trade name: Vidaza or Azadine) and 5-aza-2′-deoxycytidine (trade name: Decitabin or Dacogen) have been approved for the treatment of the myelodysplastic syndrome and other forms of blood cancers [61,62]. Another cytidine analogue that is now in clinical trials for blood cancers is Guadecitabine (SGI-110) [63]. Guadectiabine is a 5-aza-2′-deoxycytidine and 2′-deoxyguanosine containing dinucleotide that is largely resistant to degradation by cytidine deaminase [64].

On molecular level, 5-aza-cytidine and 5-aza-2′-deoxycytidine reactivate the expression of *RASSF1A* in different cancer cell lines [4,32]. Usually, treatment of cancer cells for several days with 1–10 μM of 5-aza-cytidine or 5-aza-2′-deoxycytidine leads to the reexpression of the *RASSF1A* transcript [27]. Consistently, demethylation of the *RASSF1A* promoter was also observed. Treatment of an ovarian cancer cell line with 5 μM SGI-110 (5-aza-2′-deoxycytidine containing dinucleotdide) for two days induced *RASSF1A* hypomethylation and reexpression [65]. Zebularine is yet another DNMT inhibitor and nucleoside analogue of cytidine that reactivates *RASSF1A* [60,66]. It lacks the amino group at position 4 of cytidine and also inhibits cytidine deaminases [67]. A number of studies have provided evidence that several natural compounds found in food and herbs can inhibit DNMT activity or downregulate DNMT expression (Table 1) and modulate DNA methylation of tumor suppressor genes, like *RASSF1A* or *p16/CDKNA2* [68,69]. However, there is no naturally occurring substance that function as a cytidine analogue for DNA demethylation.

## 5. Effects of Methyl Donors and Vitamins on *RASSF1A* Methylation

Folate, methionine, cobalamin (vitamin B12), betaine and choline are natural compounds that serve as natural methyl donor for the DNA methylation reaction [87,88]. These compounds function as precursors to generate S-adenosyl-methionine (SAM) which is then used as a substrate by DNMT to methylated DNA [88]. Dietary methyl donors were shown to have epigenetic effects in mice studies which showed that high maternal intake of folic acid, vitamin B12, choline and betaine can silence a transposable element through its increased methylation [89]. In breast cancer, both positive (hypermethylation) and inverse (hypomethylation) correlations with high intake of methyl donors were observed [90].

Some of these methyl donors were studied regarding their effect on the methylation level of *RASSF1A* (Table 1). It has been reported that dietary folate and alcohol intake could be associated with changes in promoter hypermethylation (*RASSF1A* and other TSG) in patients with sporadic colorectal cancer [91]. This study indicated that folate has a protective role against promoter methylation [91]. In another study, the methylation level of a panel of ten genes including *RASSF1A* in blood cells of monozygotic twins with discordant smoking habits was analyzed and the methylation index was correlated with plasma levels of folic acid, vitamin B12 and homocysteine [70]. The increased methylation index of overall promoter methylation (e.g., decreased methylation of *RARB* and *CDH1*), displayed a significant inverse correlation with plasma folic acid levels both in smokers and in non-smokers [70]. However, RASSF1A methylation levels were not significantly lower in subjects with higher plasma folic acid levels (>4.6 ng/mL) [70]. Other data for lung cancer suggested that smoking, sex, and alcohol intake had a strong influence on the methylation levels of single genes (*RASSF1A* and *MTHFR*), whereas folate intake had no significant influence on their methylation states [92]. The *methylene tetrahydrofolate reductase* (*MTHFR*) gene encodes an enzyme in the folate cycle and is important for generating the active form of folate, which is then used in the methionine cycle to synthesize the methly-donor SAM (S-adenosyl-methionine) for the DNA methylation reaction [93]. Interestingly genetic polymorphisms of *MTHFR* have been associated with an increased risk of cancer [93,94] and hypermethylation of *RASSF1A* [95,96]. Others have investigated the effect of methionine on *RASSF1A* methylation levels [71,72]. Vineis et al. examined the association between DNA methylation patterns of candidate genes and the level of methionine in the blood of lung cancer patients [72]. They report that folate levels were correlated with increased methylation of *RASSF1A* and *MTHFR*, but methionine levels were associated with decreased methylation of *RASSF1A* [72].

Vineis et al. also analyzed vitamin Bs with the methylation of *RASSF1A* and other TSG. Their data suggest that increased vitamin B12 levels are correlated with a decrease of *RASSF1A* methylation in former smorkers [72]. In breast cancer patients, the level of dietary methyl donors was correlated with the promoter hypermethylation status of *retinoic acid receptor-beta (RARB)*, *BRCA1* and *RASSF1A* [71]. There was no association with nutritional intakes and *RASSF1A* methylation, but high dietary intake of folate increased the chance of demethylation-dependent expression of *BRCA1* [71]. In another study, randomized breast cancer patients received daily supplements of co-enzyme Q10, riboflavin and niacin (vitamins B2 and B3, respectively) along with tamoxifen [97]. A significant decrease in *RASSF1A* methylation was found in patients treated with nutritional supplements compared to control patients [97]. The exact mechanism that regulates preferential modulations of DNA methylation levels of a specific set of genes by methyl donors and vitamin Bs in specific cancer types is still enigmatic [72]. Other vitamins that target DNMT are retinoic acid (vitamin A) [98] and cholecalciferol (vitamin D3) [99], but these were not tested for their effects on the *RASSF1A* methylation level in cancer cells.

## 6. Impact of Naturally Occurring Polyphenols on *RASSF1A* Methylation

Several natural occurring compounds are derivates of polyphenols including resveratrol, curcumin, genistein and epigalloctechin-3-gallate (EGCG) and these regulate DNMT activity or expression [100,101]. Some of these substances have been tested for the capacity to reactivate *RASSF1A* (Table 1). Scoccianti et al. have analyzed a group of smokers who were on a normal isocaloric diet or a diet enriched with flavonoids of green tea and soy products [102]. They analyzed the methylation level of *RASSF1A* and other regions (e.g., LINE1 with 72% methylation level) in white blood cells of smokers before and after four weeks of diet. The methylation level of *RASSF1A* was not affected by these diets, since it was rather unmethylated. However, repetitive poly-A retrotransposons (LINE1) showed a small but significant increase (1–2%) in their methylation levels [102].

The green tea polyphenol EGCG inhibits DNMTs and reactivates silenced TSG and DNA repair genes (*p16* and *MLH*1, respectively) through their demethylation in cancer cell lines [73]. Fang et al. also compared the demethylation capacity of EGCG to 5-aza-2′-deoxycytidine and showed that a treatment with 50 μM EGCG is comparable to 8.7 μM 5-aza-2′-deoxycytidine [73]. The effect of EGCG on the reactivation or demethylation of *RASSF1A* has not been reported (Table 1). Resveratrol and curcumin function as antioxidants, but also regulate DNMT activity [103]. Resveratrol has been tested for its capacity to reactive TSG in cancer cells [74,99]. It has been reported that resveratrol demethylates *RASSF1A* in women with increased breast cancer risk [74]. Women with an increased breast cancer risk were treated with trans-resveratrol twice a day for 12 weeks. Methylation assessment of four cancer-related genes including *RASSF1A* was performed on mammary ductoscopy specimens and *RASSF1A* methylation decreased with increasing levels of serum trans-resveratrol [74].

Curcumin is a polyphenol isolated from turmeric which inhibits DNMT and is a potential chemo-preventive substance [104,105]. It has also been demonstrated that curcumin (10–20 μM for 72 h) can enhance the expression level of *RASSF1A* and decrease its promoter methylation in breast cancer MCF7 cells [76]. Curcumin also reactivates *RARB* by decreasing its DNA methylation level in lung cancer cell lines [75].

Several polyphenols from the soy bean, including the isoflavones genistein and daidzein, were tested for their capacity to reactivate TSG [77,78,101]. It has been shown that these isoflavones regulate DNMT expression or inhibit DNMT activity [69,101,106]. In prostate cancer cells the demethylation effects of genistein and daidzein were compared to 5-aza-cytidine for *RASSF1A* and other hypermethylated promoters [78]. After treatment by soy isoflavones, demethylation of certain promoter regions (*GSTP1* and *EPHB2*) occurred (Table 1), but this was not observed for *RASSF1A* [78]. Vardi et al. also compared the demethylation capacity of genistein to 5-aza-cytidine in prostate cancer cell lines and showed that 40 μM genistein is comparable to 2 μM 5-aza-cytidine treatment [78]. Qin et al. treated premenopausal women daily with isoflavones through one menstrual cycle and analyzed the methylation of *RASSF1A* and other cancer-related genes in intraductal samples [77]. They reported that isoflavones induced dose-specific changes in *RARB* and *CCND2* (*cyclin D2*) methylation. High genistein levels correlated with increased methylation of *RARB* and *CCND2* [77]. Again, *RASSF1A* methylation was not significantly affected by this treatment, indicating that genistein is not involved in selective demethylation of the *RASSF1A* locus (Table 1).

Emodin is a natural polyphenol and anthraquinone derivate from *Rheum palmatum* [107]. Zhang et al. utilized 40 μM emodin to treat pancreatic cancer cells for three days and analyzed the effect on the methylation levels of *RASSF1A* [80]. They reported the demethylation of *RASSF1A*, *p16* and *preproenkepahlin* (*ppENK*) and that emodin downregulates DNMT levels (Table 1). Subsequently they showed that *RASSF1A* demethylation by 40 μM emodin is comparable to 1 μM 5-aza-2′-deoxycytidine treatment and *RASSF1A* demethylation is enhanced by co-treatment with emodin and 5-aza-2′-deoxycytidine [79].

Peperomin E is another natural polyphenol that has been utilized to study the reactivation of *RASSF1A* (Table 1). Peperomin E is a bioactive secolignan from the plant *peperomia dindygulensis* [108]. Peperomin E binds to the active pocket of DNMT1 and reduces DNMT1 levels [81]. Wang et al. treated lung cancer cells with 0.5–2 μM peperomin E for 48 h and analyzed the methylation and expression of *RASSF1A* and other TSG [81]. It was shown that the treatment with 2 μM of peperomin E resulted in the demethylation and reexpression of *RASSF1A*, *p16*, *APC* and *RUNX3* in A549 lung cancer cells [81]. Wang et al. also showed that the treatment with 2 μM peperomin E is comparable to 2 μM of 5-aza-2′-deoxycytidine in its demethylation capacity [81]. They propose that peperomin E may serve as a potential chemotherapeutic agent for non-small cell lung cancer, since it also promotes apoptosis and cell cycle arrest [81].

Dioscin is a polyphenolic component isolated from *Phyllanthus amarus* which exhibits anti-oxidative activity [109]. This substance was utilized to treat bladder cancer cell lines and to analyze its effect on the *RASSF1A* and *DAPK1* expression and methylation [82]. After 48 h of treatment with 5 and 25 μg/mL dioscin, bladder cancer cells showed induced *RASSF1A* levels and this increase was correlated with *RASSF1A* demethylation [82]. However, the impact of dioscin on DNMT expression and activity was not analyzed (Table 1).

## 7. Effects of Other Natural Compounds on *RASSF1A* Methylation

Mahanine is a carbazole alkaloid from plants (e.g., curry tree/*Murraya koenigii*) with antioxidative activity [110]. It has also been shown that mahanine inhibits DNMT activity [83,84]. Mahanine (1–3 μg/mL) was utilized to treat cancer cells and to reactive *RASSF1A* expression. *RASSF1A* rexpression was observed in prostate, breast and lung cancer cell lines [84,85]. Further data suggest that mahanine is involved in demethylation of the *RASSF1A* promoter [83].

Phenethyl isothiocyanate (PEITC) is a natural compound from cruciferous vegetables that possesses anti-cancer activities [111]. Boyanapalli et al. treated prostate cancer cells with 5 μM PEITC for 5 days and analyzed the *RASSF1A* methylation and expression levels [86]. They reported that this treatment induced *RASSF1A* expression by its promoter demethylation (Table 1). Boyanapallli et al. also showed that demethylation of *RASSF1A* by treatment with 5 μM of PEITC is comparable to 2.5 μM of 5-aza-2′-deoxycytidine [86]. Furthermore, it was shown that PEITC significantly reduced DNMT1, DNMT3A and DNMT3B protein levels [86]. However, other genomic regions have not been analyzed so far.

## 8. Conclusions

Several natural occurring substances have already been utilized to study their epigenetic activity and to analyze the effect on *RASSF1A* reactivation (Table 1). The role of methyl donors (e.g., folate and vitamin B12) on the regulation of methylation levels of *RASSF1A* should be analyzed in further detail and the precise mechanism that modulates this methylation needs to be elucidated. It has also been reported that vitamin A and vitamin D regulate DNMT expression and revert the epigenetic silencing of TSG. Thus it would be interesting to test these substances and others for *RASSF1A* demethylation and to elucidate the pathways involved in the reactivation process. Several polyphenols, like EGCG and genistein, inhibit DNMT activity and the treatment with these polyphenols lead to demethylation of certain TSG. However, methylation of *RASSF1A* was not affected by some of these compounds (e.g., genistein). In contrast, conventional demethylating agents such as synthetic cytidine analogues (e.g., 5-aza-2′-deoxycytidine) lead to reactivation of *RASSF1A*. Mahanine and peperomin E are also compounds that reactivate *RASSF1A* by inhibiting DNMT activity. The selective mechanism of some substances (e.g., methyl donors), that on the one hand side promote demethylation of certain TSG but on the other hand cause hypermethylation of other regions (e.g., retrotransponsons), remains enigmatic. Further efforts are needed to address distinct pathways responsible for selective demethylation of TSG like *RASSF1A*.

## Figures and Tables

**Table 1:** Natural compounds tested for *RASSF1A* reactivation in cancer.

Compound	Effect on *RASSF1A*	Effect on Other Genes	Mechanism	References
folate	no effect [70,71], increased methylation [72]	decreased methylation of *RARB* [70,71], *BRCA1* [71] and *CDH1* [70], increased *MTHFR* methylation [72]	methyl donor	[70,71,72]
methionine	decreased methylation	decreased methylation of *p16* and *MTHFR*	methyl donor	[72]
vitamin B12	decreased methylation	increased *MTHFR* methylation [72]	methyl donor	[71,72]
EGCG ^a^	not analyzed	decreased methylation of *p16*, *RARB*, *MGMT* and *MLH1*	inhibits DNMT ^b^ activity	[73]
reseveratol	decreased methylation	no effect on *p16*, *APC* and *CCND2* methylation	downregulation of DNMT	[74]
curcumin	decreased methylation	decreased methylation of *RARB* [75]	downregulation of DNMT	[76]
genistein	no effect	increased methylation of *RARB* and *CCND2* [77], decreased methylation of *GSTP1* and *EPHB2* [78]	inhibits DNMT	[77,78]
emodin	decreased methylation	decreased methylation of *p16* and *ppEN*K	downregulation of DNMT	[79,80]
peperomin E	decreased methylation	decreased methylation of *p16*, *APC* and *RUNX3*	inhibits DNMT activity	[81]
dioscin	decreased methylation	decreased methylation of *DAPK1*	antioxidant	[82]
mahanine	decreased methylation	not reported	inhibits DNMT activity	[83,84,85]
PEITC ^c^	decreased methylation	not reported	downregulation of DNMT	[86]

^a^ epigalloctechin-3-gallate; ^b^ DNA methyltransferase; ^c^ phenethyl isothiocyanate.

## Abbreviations

RASSF	Ras Association Domain Family
TSG	tumor suppressor gene
DNMT	DNA methyltransferase
HDAC	histone deacetylase
EGCG	epigalloctechin-3-gallate
LINE1	long interspersed nuclear element 1
PEITC	phenethyl isothiocyanate
aa	amino acids
